# From microbial diversity to functional potential using dimensionality reduction

**DOI:** 10.3389/fmicb.2026.1786397

**Published:** 2026-05-18

**Authors:** Emelia J. Chamberlain, William Boulton, Elizabeth Connors, Theodore Calianos, Jeff S. Bowman, Jessie M. Creamean, Thomas Mock, Heather H. Kim

**Affiliations:** 1 Department of Marine Chemistry and Geochemistry, Woods Hole Oceanographic Institution, Woods Hole, MA, United States; 2 School of Computing Sciences, University of East Anglia, East Anglia, United Kingdom; 3 Scripps Institution of Oceanography, University of California, San Diego, San Diego, CA, United States; 4 Department of Atmospheric Science, Colorado State University, Fort Collins, CO, United States; 5 School of Environmental Sciences, University of East Anglia, East Anglia, United Kingdom

**Keywords:** Arctic Ocean, bacteria, ecosystem function, machine learning, microbial diversity

## Abstract

The high dimensionality of microbial diversity data from ‘omics observations can be reduced using Machine Learning, with many recent studies showcasing ML utility for exploratory ecological feature finding and process prediction. Here, we compare the Self Organizing Map (SOM) dimensionality reduction method to the well-documented sample-based Principal Coordinate Analysis (PCoA) and taxa-based Weighted Gene Correlation Network Analysis (WGCNA) using near daily 16S rRNA gene amplicon sequencing data from the 2019 to 2020 MOSAiC International Arctic Drift Expedition. We then map k-means clustering outputs from each method to available metagenomes, extracting functionally distinct seasonal microbial ecotypes in the surface Arctic Ocean. Our results indicate the SOM method better represented expected seasonal transitions and identified a greater number of metabolically distinct functional groups than the more traditional PCoA ordination. Ultimately, we identified four community ecotypes with distinct taxonomic and functional cut-offs driven by seasonality, water mass, and substrate turnover, highlighting the importance of succession in functional diversity for the central Arctic Ocean. These results reinforce ML dimensionality reduction as a meaningful translator in the mining of historical amplicon datasets to address modern mechanistic questions and potentially provide ’omics informed ecotype diversity to leverage in mechanistic biogeochemical models.

## 1 Introduction

Microorganisms represent the most abundant and diverse form of life on Earth (Spang and Ettema, 2016). The tremendous taxonomic and functional diversity of microbial communities serves as a fundamental driver of ecosystem functions, marine carbon and biogeochemical cycles, and trophic energy transfer, with different species and functional groups contributing uniquely to these processes (Henson et al., 2011). Among these microbes, marine heterotrophic bacteria (hereafter bacteria) and archaea are particularly important, processing vast quantities of carbon through their complex metabolic networks. Bacteria alone respire up to 85–90% of the 50 petagrams of carbon fixed annually by phytoplankton (Field et al., 1998; Giering et al., 2014), an amount equivalent to half of global photosynthesis, making them essential players in regulating whether this carbon returns to the upper ocean-atmosphere boundary as CO_2_ or sinks to the deep ocean for centuries to millennia (Devries et al., 2012; Honjo et al., 2014; Boyd et al., 2019). However, the role diversity plays in regulating this process is not well constrained and many bacterial growth traits can vary widely among microbial groups (Li et al., 2025), with the most abundant taxa contributing little to overall rate processes (Munson-McGee et al., 2022).

Over recent decades, advances in sequencing technologies have enhanced our ability to profile bacterial communities, yielding vast, ‘omics datasets (Mock et al., 2022). As the importance of microbial contributions to climate processes becomes increasingly recognized, so too does the need to translate these complex community patterns into functional units that can inform predictive biogeochemical models (Hellweger, 2020). Machine learning (ML) has emerged as a powerful approach for reducing dimensionality across complex microbial datasets and identifying ecologically and environmentally relevant features. While all ‘omics data are rich in biological information, high-dimensional amplicon sequence variant (ASV) data are particularly suitable for ML techniques (McElhinney et al., 2022). Recent studies have highlighted this, using both supervised and unsupervised ML methodologies to distill the complexity of microbial community structure into trait-based groupings, i.e., “ecotypes” linked to specific ecosystem functions (Bowman et al., 2017) and extract the best taxonomic predictors for the key biogeochemical rate processes involved in bacterial growth efficiency, such as bacterial production (Connors et al., 2024) and oxygen utilization (Chamberlain et al., 2025).

Highlighting the field’s excitement around these methods, a flurry of recent reviews have explored why and how to apply this growing toolkit for microbial ecologists (Asnicar et al., 2023). For example, Qu et al. (2019) demonstrated how supervised classifiers can predict host phenotypes from microbiome profiles, highlighting the potential of ML to capture complex ecological patterns. Topçuoğlu et al. (2020) introduced an interpretable and reproducible ML pipeline tailored to microbiome data, addressing barriers around accessibility and transparency in ecological applications. Bowman (2021) highlighted the predictive capacity of microbial ML applications, while Hernández Medina et al. (2022) emphasized integrating environmental metadata and outlined modeling-specific considerations for microbiome data. While ML shows clear utility for microbial trait extraction, mechanistic connections between microbial community composition and ecosystem-level processes remain underexplored, despite evidence suggesting their value for quantifying ecosystem dynamics through biogeochemical modeling (Li et al., 2025). This is largely because the translation of microbial community-level patterns into functional representations suitable for, e.g., ocean biogeochemical models, presents significant methodological and conceptual challenges. As computational approaches increasingly interface with these predictive ocean models, understanding when and which ML frameworks can be effectively used to translate microbial community composition into the functional units needed for such modeling frameworks becomes increasingly relevant.

In this study, we compare and evaluate three dimensionality reduction approaches compatible with k-means clustering as a strategy to navigate the high-dimensionality challenge of bacterial data: (1) Principal Coordinate Analysis (PCoA) (Anderson and Willis, 2003), (2) Self-Organizing Maps (SOMs) (Kohonen, 2013), and (3) Weighted Gene Correlation Network Analysis (WGCNA) (Zhang and Horvath, 2005). We first employ such dimensionality reduction as k-means clustering directly on community data has the potential to impose artificial boundaries on continuous ecological gradients. These specific methodological representatives were chosen because they are well established in the literature and highly interpretable (not black-box prediction), while still providing a gradient of analytical complexity, capturing different levels of ecological structure. While all three approaches account for the underlying structure embedded in high-dimensional microbial datasets, they differ significantly in complexity, assumptions, outputs, and interpretability. Each method offers distinct advantages: PCoA provides a traditional statistical ordination baseline that summarizes dominant variation among samples by projecting it into a lower-dimensional space (Legendre and Legendre, 2012). SOMs provide a ML and non-linear alternative, training an unsupervised neural network via competitive learning algorithm to identify emergent groupings (Kohonen, 2013; Oja et al., 2003) WGCNA resolves taxon-level modules by constructing statistical weighted correlation networks to identify discrete modules of co-varying taxa, offering a more taxa-driven framework suitable for hypothesis-driven ecological analysis (Zhang and Horvath, 2005).

We test these approaches using a multi-seasonal bacterial dataset from the surface waters of the central Arctic Ocean, in a region where climate warming is occurring at almost four times the global average rate (Rantanen et al., 2022). This ocean represents an ideal testbed for comparing dimensionality reduction methods due to its extreme seasonal bacterial shifts (Priest et al., 2025) and the critical roles these communities play in carbon cycling processes that could significantly impact Arctic amplification (Edwards et al., 2020). The rapid environmental transitions in the Arctic have the potential to release previously sequestered carbon (Schuur et al., 2015), while fundamentally altering bacterial community structure and function (Smith et al., 2023). Identifying which translational approach best captures these complex ecological patterns in high-dimensional Arctic bacterial datasets is therefore essential for improving future model predictions of regional and global climate responses mediated by microbial feedbacks.

## 2 Materials and methods

### 2.1 Sample selection and collection

The Multidisciplinary drifting Observatory for the Study of Arctic Climate Expedition (MOSAiC; 2019–2020) was an interdisciplinary year-long drift experiment in the central Arctic Ocean that created a wealth of ecological (Fong et al., 2024) and genomic (Mock et al., 2022) data which we have leveraged for this study. From the publicly available MOSAiC 16S rRNA gene amplicon sequencing dataset (Chamberlain and Bowman, 2022), a subset of 215 suitable surface seawater samples were selected and assessed. These were collected as close to daily as possible (average sampling time 15:00 UTC) between 10/29/2019 and 09/18/2020 over the course of the MOSAiC drift, covering a 10° latitudinal gradient 89.1°–79.1° across the Amundsen and Nansen Basins (Fong et al., 2024). Data gaps exist where project personnel were not on board (12/13/2019–02/20/2020) and during periods of ship relocation (07/31/2020–08/22/2020). Samples were collected from the underway seawater system (RV Polarstern; 11 m inlet), with highly comparable community structure results to samples from similar depths collected using a CTD Rosette (Chamberlain et al., 2025). Additionally, 18 of the underway sampling dates matched with whole metagenome data collected at either 10 or 11 m depth from the CTD rosette (Mock et al., 2022). Ancillary environmental data (surface temperature and salinity) were collected continuously from the underway system and corrected as described in Schulz et al. (2024). Chlorophyll-a concentrations were collected and filtered at the same time as collections for microbial community structure and are described by Hoppe et al. (2023).

### 2.2 DNA sequencing and metabolic function

Sample and data processing for water column bacterial and archaeal community structure (16S rRNA gene amplicon sequencing) is described in detail by Chamberlain et al. (2025). Briefly, 1 L of underway seawater was filtered through a 25 mm 0.2 μm Pall Corporation Supor membrane filter and frozen (–80 °C). DNA was extracted using a ThermoFisher Scientific Kingfisher*™* Flex MagMax Microbiome Ultra Nucleic Acid extraction kit and sequencing for the amplified V4 region of 16S rRNA gene took place at Argonne National Laboratory on the Illumina MiSeq platform (universal primers 515F and 806R; Walters et al., 2016). Illumina reads were cleaned using dada2 (Callahan et al., 2016) and classified using PAthway PRediction by phylogenetIC placement (paprica v0.7.0; Bowman and Ducklow, 2015) with a database derived reference tree (Genbank RefSeq; Haft et al., 2018). Point of placement was additionally used to perform metabolic inference estimating genetic characteristics, including genome size, 16S rRNA gene copy number, and GC content (Erazo et al., 2021) and doubling time (gRodon; Weissman et al., 2021). Note, doubling times from codon usage reflect theoretical growth rates, and neglect environmental conditions, serving only as a theoretical maximum to compare between samples. Additionally, taxonomic associations from paprica represent an ASV’s closest relative among the published Genbank RefSeq completed genomes and don’t always indicate the presence of an exact strain. Estimated 16S rRNA gene copy number was used to normalize read counts prior to calculations of relative abundance. Singlton ASVs were not included, and data was normalized using the Hellinger Transformation (performed in “vegan”; Oksanen et al., 2015), making it more suitable for downstream clustering analyses with assumptions of Euclidean distances (Legendre and Gallagher, 2001).

The extraction of DNA from the 18 metagenomes is the same as described in Boulton et al. (2025) (Boulton et al., 2025). Seawater was filtered through 0.22 μm Sterivex filters, and DNA was extracted using the Qiagen PowerWater DNA kit (Qiagen N.V., Hilden, Germany), following the Qiagen DNeasy Power Water SOP v1. Samples were sequenced on an Illumina NovaSeq S4 device, with 151 base pair paired-end reads, using the Illumina regular concentration library protocol. Metagenomes were assembled, annotated, and reads mapped by the JGI Metagenome Annotation Pipeline (Clum et al., 2021). To assess potential metabolic function within the 18 metagenomes, we calculated the relative abundance of COG categories (Tatusov et al., 2000), based on transcripts per million (tpm). The tpm abundance of each COG category was then calculated based on the normalized combined coverage of each CDS containing a COG annotation.

### 2.3 Machine learning and statistical analysis

All analyses were conducted in R (R Core Team, 2023). The PCoA was constructed using the “vegan” package (Oksanen et al., 2015) to provide a low-dimensional projection of samples based on Euclidean distances which was separated into distinct clusters using the unsupervised ML k-means algorithm. The final selection of k was determined using both the elbow method, or inflection in within-clusters sum of squares (WSS), and the Silhouette method. While the elbow method identifies the point of diminishing returns in terms of cluster compactness, silhouette scores also account for between-cluster separation. Experimentally testing k values around the perceived optimums (experimental process described in Supplementary Text 1), we compared how well clusters separated in the first two ordination axes and used a post-hoc Permutational Multivariate Analysis of Variance (PERMANOVA) test to confirm that clusters were statistically significant.

The unsupervised SOM model was constructed using the “kohonen” package (Wehrens and Kruisselbrink, 2018). Grid parameters were experimentally selected, while aiming for an optimal distribution of samples assigned to each map unit (Supplementary Figure 1). The final map was constructed using a 4 × 4 toroidal grid with hexagonal map units and a decay scaling alpha from 0.05 to 0.01. Unit assignment was based on Euclidean distances and clustering also took place via k-means. This is a similar method to what was used on a full water column MOSAiC dataset in Chamberlain et al. (2025) but with a unique SOM construction and sensitivity due to the differing resolution of input data (215 surface underway vs. 693 samples underway and CTD samples, 0–4,000 m). Resulting differences are described in detail in Supplementary Figure 2. Ultimately, through the combination of dimension reduction (PCoA or SOM) and k-means clustering, each sample was assigned to unique community compositional clusters which we were then statistically compared to metabolic inference and environmental parameters using Kruskal Wallis and *post hoc* Wilcoxon signed-rank tests (rstatix package; Kassambara, 2023).

The WGCNA analysis was constructed using the “WGCNA” package (Langfelder and Horvath, 2008) and a hybrid network construction through the application of k-means clustering instead of the standard hierarchical clustering and dynamic tree cut, inspired by the framework described in Botía et al. (2017). A soft-thresholding power of 8 was chosen based on the scale-free topology fit index (*R*^2^ > 0.8) and mean connectivity profiles. An adjacency matrix was computed using a signed network derived from Pearson correlations, followed by transformation into a topological overlap matrix to capture shared patterns of co-abundance among ASVs. To detect modules of co-varying ASVs, we applied k-means clustering to the TOM-based dissimilarity matrix. Following module detection, each module was summarized by its eigengene—the first principal component of the ASV profiles within the module. These module eigengenes were then correlated with functional metadata (estimated 16S rRNA gene copy number, doubling time, genome size, GC content, and total chlorophyll-a concentration) and environmental parameters (temperature, salinity, and location) to identify ecologically relevant modules. Module-trait relationships were visualized in a heatmap with both correlation coefficients and *p*-values. Similar modules were merged using a correlation-based threshold of 0.7 to reduce redundancy. To assess the importance of individual ASVs within each module, we calculated module membership scores (kME), defined as the Pearson correlation between each ASV’s abundance profile and the module eigengene. These scores were used to rank ASVs by their centrality within a module, allowing identification of the taxa with the strongest association to overall module structure and likely ecological influence.

To provide further comparison across methods using alternative methods from those used in clustering, a non-metric multidimensional scaling (NMDS) ordination was constructed from the Bray–Curtis dissimilarity matrix of Hellinger transformed ASV relative abundances (vegan package; Oksanen et al., 2015) and correlated with basic environmental parameters representative of season and location.

## 3 Results

The underway surface water amplicon dataset contained a total of 5,135 unique ASVs across 215 samples. The PCoA and SOM models, which are robust to zeros and outliers, used all samples and sequences for model construction. The WGCNA correlation network approach, which is sensitive to outliers and rare abundances, has a standard data QC and selection procedure applied during analysis (Langfelder and Horvath, 2008). This removed 3 of the original samples and retained only the most abundant 1,322 sequences for clustering. Ultimately, K-means clustering yielded 3 PCoA (Supplementary Figure 3), 5 SOMs (Supplementary Figures 1, 3), and 4 WGCNA (Supplementary Figure 4) unique microbial community clusters respectively.

**FIGURE 1:**
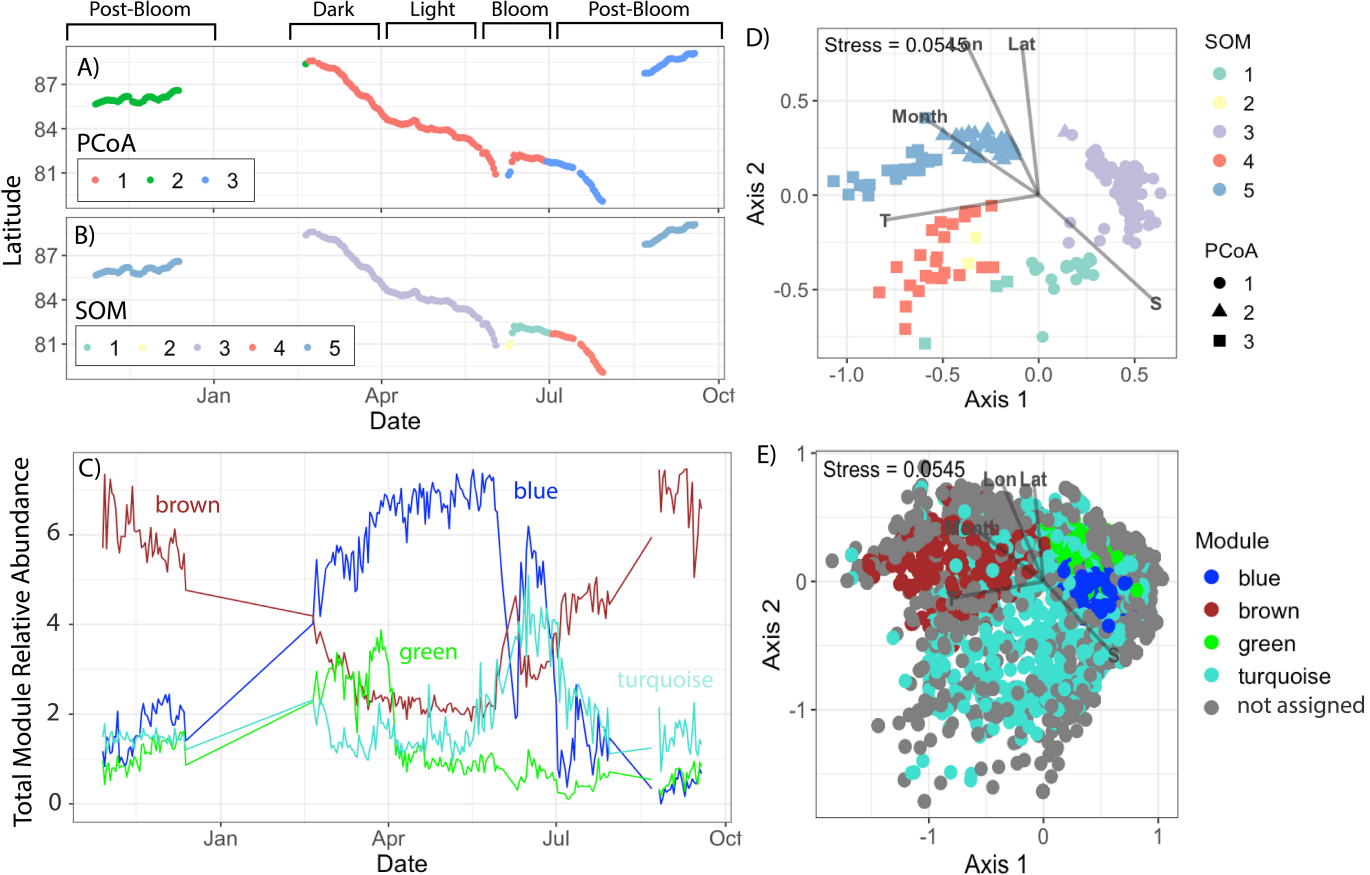
Clustering output and spatio-temporal distributions during MOSAiC. PCoA **(A)** and SOM **(B)** clusters identified distinct seasonal shifts in bacterial community structure throughout the MOSAiC Drift, here visualized as latitude. In **(C)**, the relative abundances of all ASVs identified to each WGCNA module were summed for each sampling date. Environmental separation among clusters was significant [indicated by the gray arrows for latitude (Lat), longitude (Lon), numerical month, temperature (T), and salinity (S)] when samples **(D)** and ASVs **(E)** were visualized in the first two axes of an NMDS ordination created from the bray dissimilarities of Hellinger transformed 16S ASV relative abundances. The NMDS stress value, indicating ordination fit, is listed in the top left-hand corner. In **(D)** samples are colored by SOM and shaped by PCoA cluster assignment. In **(E)** ASVs are colored by module assignment. Gray points represent ASVs with total relative abundances too low to be included in the WGCNA analysis.

All clustering methods displayed evidence of seasonal community succession, but there was substantial variance between the temporal cut-offs for each methodology (Figure 1). Projecting the underlying data structure of both the samples (Figure 1D) and individual ASVs (Figure 1E) using a comparative projection method (NMDS ordination), separation in the ordination space was most closely aligned with the SOM segmentation (samples) and WGCNA modules (ASVs). Numerical month (*r*^2^ = 0.56), temperature (*r*^2^ = 0.66), salinity (*r*^2^ = 0.70), latitude (*r*^2^ = 0.65), and longitude (*r*^2^ = 0.79) were all significant explanatory variables (*p* < 0.001) in the NMDS ordination (Figures 1D,E). The distribution of values for these associated environmental variables and estimated genetic traits are presented in the supplemental material for each PCoA (Supplementary Figure 5) and SOM (Supplementary Figure 6) cluster. Pearson correlation coefficients indicate a significant relationship with all environmental variables and genetic traits (*p* < 0.05) except for the brown module which was not significantly correlated with genome size (*p* = 0.08) turquoise module which was not significantly correlated with Temperature (*p* = 0.6), Numeric month (*p* = 0.5), and % GC content (*p* = 0.8) (Supplementary Figure 7).

**FIGURE 2:**
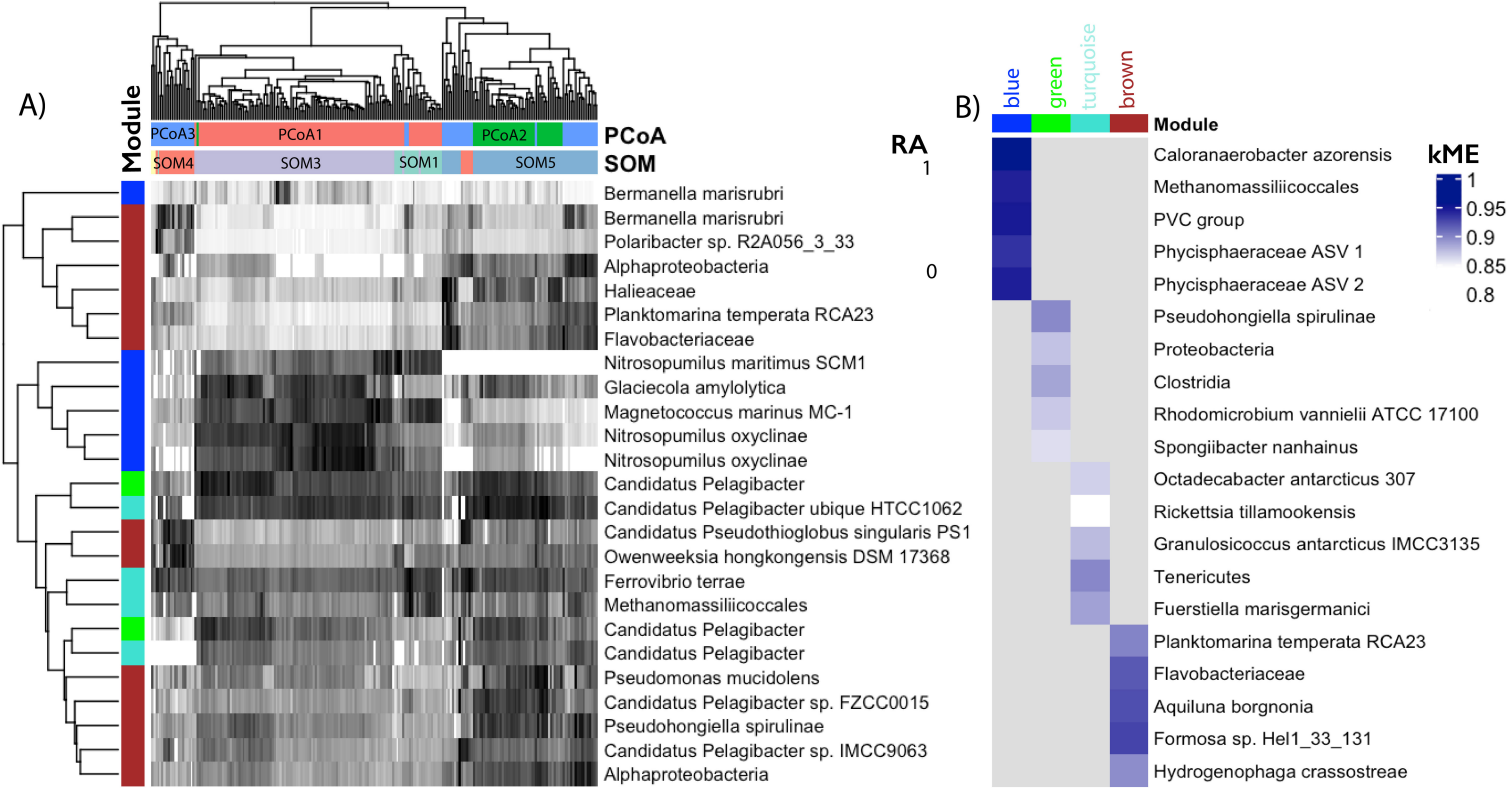
Abundant taxonomy of ML clusters and top WGCNA module membership. **(A)** Heatmap of Hellinger transformed relative abundance (RA, scaled 0–1 via z-score) for the 25 most abundant ASVs across all samples. Columns are annotated by PCoA and SOM cluster assignments, and rows are colored by WGCNA module membership. Both were clustered using euclidean distances. **(B)** Heatmap showing the ASVs with highest module membership (*k*ME) for each WGCNA module, with color labels matching the rows in panel **(A)**.

**FIGURE 3:**
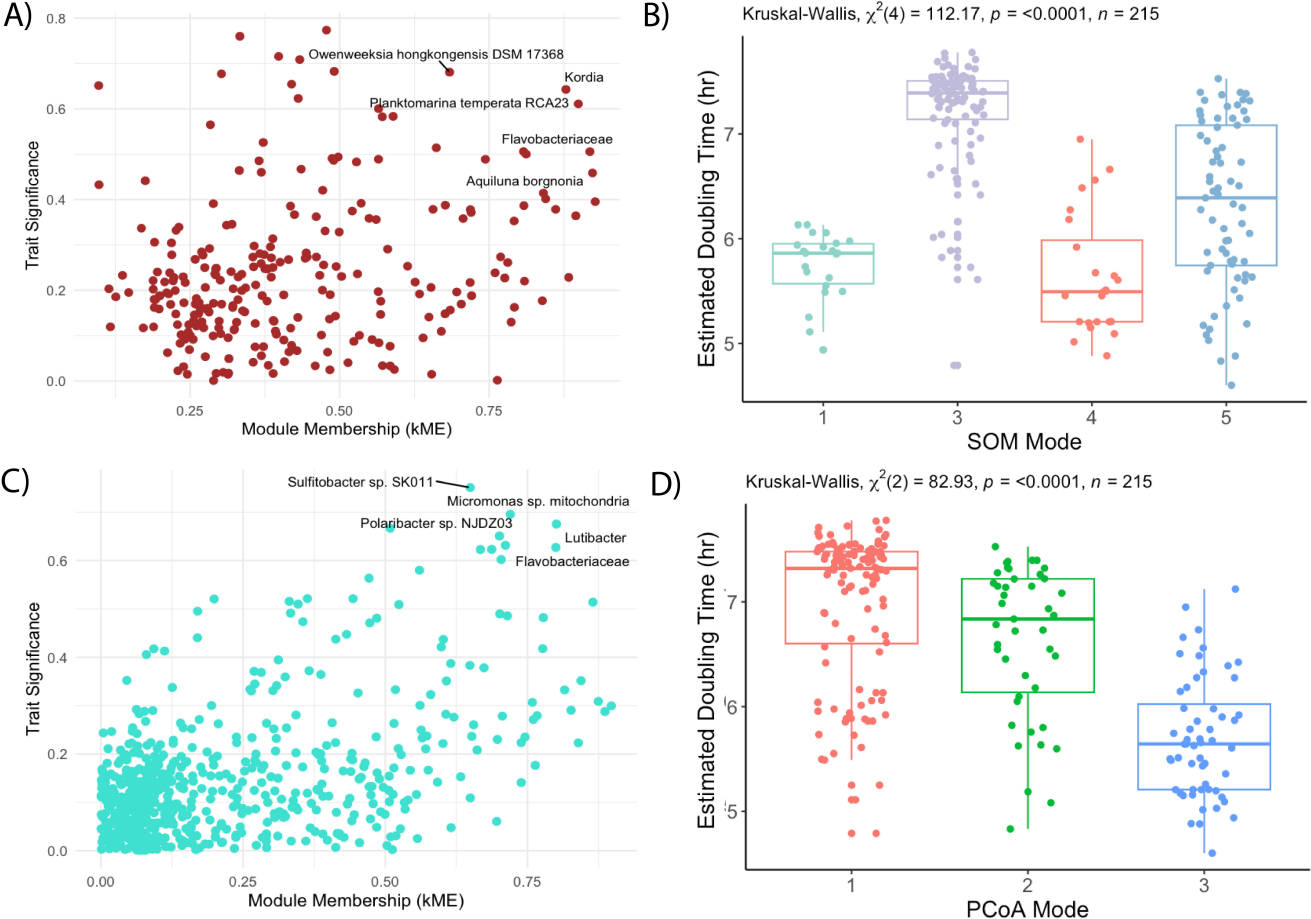
Clusters of orthologous groups (COG) enrichment across sample clusters. Top 25 most abundant COG (Clusters of Orthologous Groups) assignments using Pfam abundance (tpm) across all samples. Columns represent the 15 sample days where surface water metagenomic analyses aligned with underway 16S sampling. Columns are color annotated by PCoA and SOM cluster assignments as well as the dominant WGCNA module for that date. Rows correspond to COG assignments with 4 containing functional genes enriched in multiple COG groups simultaneously. Abundance values were normalized (tpm) and scaled from 0 to 1 using z-score calculations for plotting. Hierarchical clustering was applied to both rows and columns.

**FIGURE 4:**
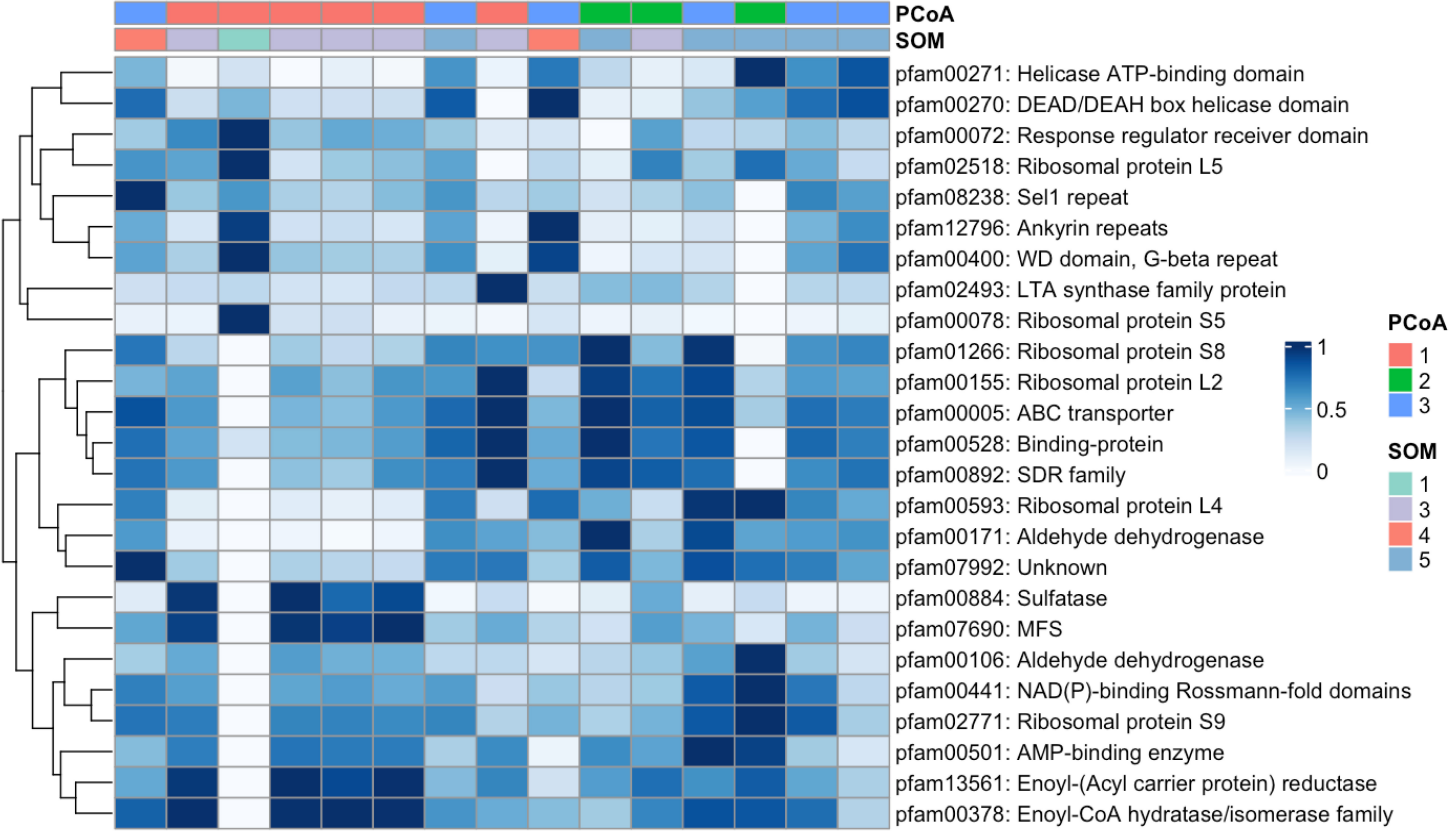
Codon-usage derived growth rates across clusters and highly correlated taxa. Growth rate is represented here by the gRodon estimated mean doubling time in hr, where a lower doubling time indicates faster growth. **(A)** Scatterplot of trait significance (doubling time) and module membership (kME) for all ASVs in the Brown module. **(B)** SOM clusters compared to doubling time. **(C)** Scatterplot of trait significance (doubling time) and module membership (kME) for all ASVs in the Turquoise module **(D)** PCoA clusters compared to doubling time. In **(A,C)**, the top five significantly correlated and high kME ASVs are labeled by closest reference genome. In **(B,D)**, the horizontal line of each boxplot indicates the median, the box spans the first to the third quartile, the whiskers indicate minimum and maximum values, and the dots indicate all values.

Seasonal cutoffs between most clusters were reinforced by clear taxonomic and genetic shifts. SOM5, for example, is present in both late Fall 2019 and early Fall 2020 and was enriched in ASVs assigned to taxa in the Brown module, including some of the top module members (e.g., *Planktomarina temperata*, and an unidentified Flavobacterium ASV; Figure 2B). Most of these top taxa represent copiotrophs associated with the rapid colonization and degradation of organic material (Dithugoe et al., 2023). SOM5 samples were additionally enriched in carbohydrate, amino acid, and lipid transport genes (Figure 3). The PCoA analysis however, distinguished two clusters during the fall periods (PCoA2 in Fall 2019 and PCoA3 in late Summer/early Fall 2020), with significant environmental drivers of water mass salinity and temperature (Figure 1D). The primary taxonomic difference between these clusters were the additional enrichment in ASVs assigned to *Bermanella marisrubi, Owenweeksia hongkongenesis*, and *Polaribacter* sp. (Figure 2A) and cell motility/translational genes (Figure 3) in PCoA3. However, these features were also prominent in the additional SOM cluster of SOM4 which is only present in July 2020 (Figure 1) and also marked by significantly lower mean doubling times (Figure 3B), higher mean 16S copy numbers, and higher mean genome sizes (Supplementary Figure 6). While the July 1 shift between PCoA2 and 3 coincides with the onset of Brown Module dominance, the taxa with both high kME and significant negative correlation to mean doubling time (Figure 4) align exactly with the topmost abundant taxa in SOM4. The Brown Module then reaches its abundance peak with the transition to SOM 5.

During early summer (June 2020), the SOM (SOM1) and WGCNA (Turquoise Module) clustering methods capture additional community complexity which was not captured in the PCoA (Figure 1). Despite being the module with the most ubiquitous background taxa (Figure 1E), the Turquoise module was significantly (*p* < 0.001) negatively correlated with mean doubling time, with its top kME and correlated taxa strongly related to phytoplankton groups (e.g., *Micromonas* mitochondrial DNA) or associated heterotrophs known for exploiting sudden pulses of organic matter (e.g., Polaribacter, Lutibacter). The Turquoise module was also the most significantly correlated with chlorophyll-a concentration (*p* < 0.001; Supplementary Figure 7) and had several high kME ASVs assigned to either phychrophilic biofilm producers or algal associates/pathogens. These functional changes in genetic information were captured with in the segmentation of SOM1, which had high chlorophyll-a concentrations, the second lowest mean doubling times, second highest mean genome size, and a low 16S copy number (Supplementary Figure 6). However, this SOM cluster was also composed of highly abundant ASVs assigned across the Blue, Brown, and Turquoise modules, which were all relatively abundant during this seasonal transition as well (Figure 1). While there was only one matching metagenome sample for this period, it was enriched in genes related to the mobilome, cell division, and transcription (Figure 3). This resulted in a unique bloom-associated community which quickly grew in abundance during this time.

The winter-spring period of the dataset (March–June 2020) was dominated by PCoA1, SOM3, and the Blue WGCNA Module (Figure 1). Abundant taxa during this time were primarily slow growing chemoautotrophs (Figure 2), with available metagenomes showing enriched genes for such complex metabolisms (Figure 4). Both PCoA1 and SOM3 had a high mean estimated doubling time (Figure 3), but with a large sample spread. This could be due to the additional intraseasonal taxonomic community highlighted by WGCNA in the Green module prior return of sunlight at the end of March (Figure 1). Prevalent only in the dark period, the Green module was made up of a unique selection of pelagibacterales taxa, however the module generally followed the same trait-relationships as the more abundant Blue module species (e.g., positive correlations with estimated doubling time and % GC content and negative correlations with Chlorophyll concentration, genome size, and 16S rRNA gene copy number; Supplementary Figure 7).

## 4 Discussion

### 4.1 Identifying seasonal microbial ecotypes for the surface Arctic Ocean

Taking the summation of information on cluster cut-offs, their external environmental drivers (e.g., season), internal community composition, and inferred and measured functional potential, we suggest 4 seasonal microbial ecotypes for the surface Arctic Ocean with distinct parameterizable functional traits (Table 1). To qualify as a unique functional ecotype, inferenced functional estimates (e.g., doubling time, genome size) had to be statistically significant from all other clusters within the same methodology (PCoA/SOM) or significantly correlated with at least 2 physiological variables (WGCNA), contain a distinctive community composition from the dominant clusters temporally before and after, and have estimated functional distinctions (Table 1) validated by available metagenome observations. Ecotypes were described by the timing which the representative clusters meeting the above criteria were present within the time series.

**TABLE 1:** Functional summary of surface ocean Arctic ecotypes.

Description	Growth rate estimate	Estimated genome size	Key taxonomic representatives	Enriched cellular functions	ML clustered output representatives
Bloom associated	Fastest	Large	Polaribacter, Suliftobacter	Motility and cell division	SOM1, Turquoise module
Post-bloom	Fast	Large	Flavobacteria, general	Carbohydrate and lipid processing	PCoA3, SOM5, Brown module
Winter/dark	Slowest	Streamlined	Various Gammaproteobacteria	Energy production and conversion	Green module
Spring/light	Slow	Streamlined	*Nitrospulimus* sp., Pelagibacter, Phycisphaeraceae sp.	Defense mechanisms, inorganic ion transport	PCoA2, SOM3, Blue module

The summer Bloom-Associated Ecotype rapidly increases its abundance during the period of the pelagic phytoplankton bloom, representing a shift in carbon source utilization and increased microbial growth rates. This Ecotype is represented in our dataset during mid-late June (Fong et al., 2024) by SOM1 (lowest estimated doubling times, high enrichment of cell division genes) and the WGCNA Turquoise Module (high kME of phytoplankton associated taxa).

The summer Post-Bloom Ecotype follows directly after the prior Bloom-Associated and represents a still fast-growing microbial community with a unique taxonomy and greater capacity for organic matter processing that indicates it should be recognized as its own functional group. In our dataset, this Ecotype is represented by PCoA2/3, SOM4/5, and the Brown Module. It is possible that the high kME taxa in the Brown Module that were significantly correlated with low doubling time and tracked the most abundant taxa in the distinctive SOM4 could also be considered its own ecotype. However, most differences between these SOMs were environmentally driven (Supplementary Figure 5) and the estimated genetic trait differences were not fully reflected in the select available metagenomes. More measurements would be required to functionally separate these two groups.

The Winter/Dark Ecotype represents the appearance of the low abundance Green module in early spring and is the only defined ecotype not to be represented across multiple clustering techniques. We still include it here, however, due to the module containing multiple unique structural features. Its most abundant ASVs (Figure 2A) were assigned to the slow growing and genomically streamlined Pelagibacterales order (Tucker et al., 2021), which were also highly abundant in the Blue module and are common in the Arctic winter and spring upper water column (Chamberlain et al., 2025). Pelagibacterales are also uniquely distinguished by their high internal diversity—even within subclades, the micro-diversity of this species is extremely high, allowing them to track subtle shifts in environmental niches across the world’s oceans (Delmont et al., 2019). It is therefore not surprising that they are highly abundant across multiple community clusters as there is likely greater speciation not captured in the reference genomes used here for taxonomic assignment. The ability to capture meaningful ecological differences and functional separation even without perfect taxonomic assignment highlights this methods robustness and is one of the primary benefits to using segmentation at the ASV-level.

The primary environmental deviation for the Green Module is an opposite correlation to Latitude, potentially indicating oceanographic partitioning at this time—although the decrease in Green module relative abundance occurs slightly after the Polarstern drifted across the Gakkel Ridge (Schulz et al., 2024). Additionally, the top kME taxa for this module (Figure 2B) are primarily specialized in breaking down recalcitrant carbon and their genomic representatives all contain at least one mechanism for boosting ATP yields during periods of electron scarcity (e.g., rhodopsins and other bacterial photosystems, motility). This indicates their suitability for surviving the scarcity period of polar night and adapting early to the return of the sun in early spring, as seen in the overwintering strategies of Arctic phytoplankton and sea-ice algae (Kvernvik et al., 2018; Hoppe et al., 2024).

The Winter/Light Ecotype (SOM3, PCoA2, Blue module) on the other hand is driven by phytoplankton associated and chemosynthetic taxa (Figure 2B), which were likely brought to the surface from deeper in the water column due to intensified mixing at this time (Chamberlain et al., 2025) and representing a total functional shift that is best reflected in the most abundant ASVs in SOM3 (Figure 2A). SOM3 is also enriched in heterotrophic metabolism gene indicators, as well as defense mechanism genes—which would reflect the growing number of Spring phytoplankton and potential grazing activities by mixotrophic dinoflagellates, which were also brought to the surface and highly abundant at this time (Chamberlain et al., 2025).

Annually recurrent microbial ecotypes have been observed in other Arctic Ocean time series (Priest et al., 2025), due to strong seasonal selection pressures (Wietz et al., 2021). The layered functional organizations offered by our multi-method comparison may further inform ongoing debates over microbial functional redundancy, a concept used to explain the stability of many microbial processes despite high individual species turnover (Louca et al., 2018) The distinct temporal windows occupied by the SOM clusters and WGCNA modules (Figure 1) and clear shifts in community composition coinciding to measurable changes in dominant metabolic function (Figure 4), support studies emphasizing a tight coupling between taxa and function (Galand et al., 2018) and limited redundancy at ecologically relevant scales of environmental change (Larkin et al., 2025). As has been found in other study systems (Wilson et al., 2021), these methods revealed a layered picture of community succession. Seasonal shifts in community structure and overall function were captured by sample-based clustering (SOMs/PCoA) as the entire assemblage shifted coherently, but inter-seasonal responses to substrate pulses depended on specific, and not fully redundant, functional guilds that only the ASV-level co-occurrence WGCNA analysis was able to extract. By building a signed correlation network among individual ASVs that co-vary across samples, the modules emerge from shared temporal dynamics, not simply relative abundance. This explains why this method was more sensitive to dynamic environmental shifts—allowing it to better resolve fine-scale resource niches.

### 4.2 Environmental sensitivity and other dataset caveats

The strong correlations of longitude and salinity in the NMDS are notable, but not unexpected. Longitude (and latitude) represent the large-scale oceanographic gradients shaping community composition. Salinity (and temperature) are related to these gradients but also impacted by seasonal ice melt—which created critical thresholds for community structure to shift as recognized across our statistical and ML clustering (Table 1). The PCoA was the least sensitive to these finer environmental shifts, recognizing only one community cluster both during summer melt and in fall 2020 when MOSAiC drifted into relatively fresher Transpolar Drift influenced waters (Schulz et al., 2024). Meanwhile, the period between June–September (summer melt season) had three distinct co-occurrence modules (Brown, Turquoise, and Blue; Figure 1C) and two unique SOM communities (SOM1 and SOM4; Figure 1B), indicating greater sensitivity of these methods to recognizing fine scale environmental and/or seasonal shifts in community structure. This advantage likely stems from the underlying data structure. PCoA axes are continuous and dominated by highly abundant taxa (Legendre and Gallagher, 2001). Rare ASVs get compressed near the origin within the original ordination, meaning their influence is likely to be under-represented (Shade et al., 2014). While this projection is useful in analyses specifically targeting a balanced representation of the community, our results indicate this may come at the cost of functional sensitivity and support other studies which have highlighted a mismatch between abundance and influence (Munson-McGee et al., 2022).

One result not yet discussed is the *n* = 2 segmentation of SOM2. Both samples were from a period of lower latitude transit through the marginal ice zone and likely represented a brief spatial disruption in temporal community succession. This scenario is also reflected in both the PCoA, which clustered these samples with the later dominant PCoA3 (Figure 1B), and WGCNA, which showed a brief inversion in the dominance of the Blue and Brown modules at this time (Figure 1C). These two samples are either true outliers in the dataset and the SOM map grid was the most sensitive projection, or it was a cut-off induced by insufficient grid-size or misinterpretation of map proximity (Kohonen, 2013). Given that during the grid size sensitivity analysis, these samples clustered with what we consider SOM1 using a grid size was 3 × 3, and SOM4 (nearest neighbor, Figure 1D) using the same grid size of 4 × 4 but choosing a k of 4, this is likely the case (Supplementary Figure 1). Because k-means clustering assumes clusters of equal variances, non-balanced clusters can result in unduly isolated, low-variance clusters (Jain, 2010). While SOM2 and SOM4 were not significantly different in most genetic parameters, there was some significant environmental variation and SOM2 did display a significantly higher GC content (*p* = 0.0062; Supplementary Figure 5). However, with only *n* = 2, we were not confident in these relationships and SOM2 was not considered functionally unique for the purposes of identifying Arctic Ocean microbial Ecotypes.

When discussing functionality, it is important to acknowledge that our analysis here is largely derived from community composition and metabolically inferred genetic profiles from amplicon data (Bowman and Ducklow, 2015), which can be biased by the available reference genomes. While this is not a measurement of functionality directly, it can still provide a valuable means of approximating metabolic potential—bridging the gap when more cost-intensive metagenomic or metatranscriptomic data are unavailable (Erazo et al., 2021). Given that the paprica output from this dataset produced functionally consistent patterns with our, albeit less temporally resolved, metagenomic observations, our study offers a cost-effective and scalable pathway to link microbial diversity with potential ecosystem function. This is especially important in data poor regions like the central Arctic Ocean where large temporal and spatial gaps exist (Mock et al., 2022). However, we do emphasize that these inferences (e.g., doubling times and genome size) represent potential and not realized function. Future work integrating multi-omics (e.g., higher resolution metagenomic or transcriptomic) and paired true biogeochemical rate measurements (e.g., Connors et al., 2024) to validate and refine amplicon-based predictions would be ideal.

### 4.3 Perspective on integrating data-driven ecotypes into mechanistic biogeochemical models

A central challenge in ocean modeling is representing the immense diversity of marine bacteria (Séférian et al., 2020). Our results demonstrate that segmentation approaches provide an efficient solution to the challenge of scaling between individual taxa and the bulk ecosystem compartments often used by process-based biogeochemical models by creating functionally meaningful intermediate units of complexity. We identified four distinct ecotypes in the surface Arctic Ocean, each characterized by clear environmental drivers and seasonal patterns. Two ecotypes dominated during winter and spring periods characterized by low light conditions, while two others were tightly coupled to the timing of summer phytoplankton blooms (Table 1). These ML-derived ecotypes represent interpretable ecological units ideal for model integration because they consolidate key physiological and functional attributes including growth strategies (16S copy number), metabolic potential (COGs), and salinity tolerance. Moreover, these approaches generate ready-made state variables whose parameters, such as maximum growth rates, temperature-salinity optima, and substrate preferences, can be constrained from multi-omic datasets, eliminating dependence on outdated single-species culture estimates. Recent studies have demonstrated significant improvements achievable through incorporating such bacterial trait structures (Zakem et al., 2025).

We propose treating each ML derived ecotype as an emergent functional unit with parameter values derived from ensemble trait measurements (e.g., median maximum growth rate, half-saturation constants). Because these ecotypes maintain clear relationships with environmental covariates such as salinity, temperature, and longitude, their abundances can be dynamically adjusted using environment-dependent growth and loss terms. This approach adds adaptive realism to models without increasing computational complexity. In doing so, the selection of appropriate segmentation methods is critical. The clear difference between our ordination approaches highlights the necessity of employing non-linear, ML dimensionality-reduction techniques when deriving model compartments. While traditional axis-based ordinations (PCoA) grouped samples by water mass characteristics, they failed to capture the seasonally coupled temperature-salinity niches that more sophisticated methods like SOM and WGCNA successfully identified. Integrating comparable ML segmentation within model-data assimilation frameworks could substantially improve state estimation and parameter optimization (Kim et al., 2021, 2022), ultimately strengthening constraints on predicted bacterial feedback to ocean carbon cycling.

## 5 Conclusion

Statistical dimensionality reduction provides a workable bridge between microbial surveys and the coarse compartments required by biogeochemical modeling. Our results indicated that functional variation and shifts in species interaction were accurately captured by k-means segmentation—notably paired with SOMs—and we were able to identify potential “functionally coherent” groupings which crossed taxonomic boundaries (e.g., Pelagibacterales, Flavobacteria) and embodied distinct variations in the genetic traits which are most useful for predictive model parameterization. The utility of compartmentalizing the community in this way is that it goes beyond “size-class” based trait extraction under the argument of functional redundancy and still allows community-based groupings when taxonomic succession does matter. Furthermore, testing our workflow on amplicon sequencing derived ASVs demonstrates how these algorithms could be used to combine and mine previously generated legacy data for new ecological insight. Amplicon sequencing has long been the more widespread and affordable methodology compared to the richer but scarcer metagenomic and transcriptomic records. However, this work could still be improved—particularly in the Arctic, where data is scarce—by producing more *paired* ‘omics datasets for such dimensionality reduction methods to get better predictive insights into the mechanisms behind biogeochemical parameters of interest in a rapidly changing ocean.

## Data Availability

Publicly available datasets were analyzed in this study. The 16S rRNA gene amplicon sequence data are available through the National Center for Biotechnology Information (NCBI BioProject #PRJNA895866), with metadata available through the Arctic Data Center (https://arcticdata.io/catalog/view/doi%3A10.18739%2FA2CC0TV5X). The raw metagenomic data are also available through the NCBI (BioProject #PRJNA1160706) (Boulton et al., 2025). Data QC and ML code pipelines are available upon request from the corresponding authors.
