# Supplementary material for: From microbial diversity to functional potential using dimensionality reduction
Source: Front Microbiol. 2026 May 18;17:1786397. doi: 10.3389/fmicb.2026.1786397 (PMC13224340; doi:10.3389/fmicb.2026.1786397)
Supplement: Supplementary file 1 [file Data_Sheet_1.pdf]

Supplementary Material

1 Supplementary Text

1. K-value selection across all models was determined using the visualized statistics of within-clusters sum of squares (WSS) and average silhouette width. A perceived optimal k was chosen by first selecting the number of clusters that most accurately represented the inflection point of the Elbow Plot (WSS vs. k; e.g. Supplementary Figure 1 a1, b1,c1; Supplementary Figure 2a; 2d; Supplementary Figure 3c) – indicating the point where adding additional clusters does not result in a significant drop in within-cluster variance. This was then cross checked with a Silhouette analysis to evaluate cluster quality, where a higher silhouette width for a given k indicates greater cluster cohesion. The average silhouette width across all samples for each k (Supplementary Figure 1 a2, b2,c2; Supplementary Figure 2b; 2e; Supplementary Figure 3d) was plotted to assess values around the perceived optimal k from the Elbow Plot. The final k-value was chosen as the local maximum in silhouette score from these values.

2 Supplementary Figures

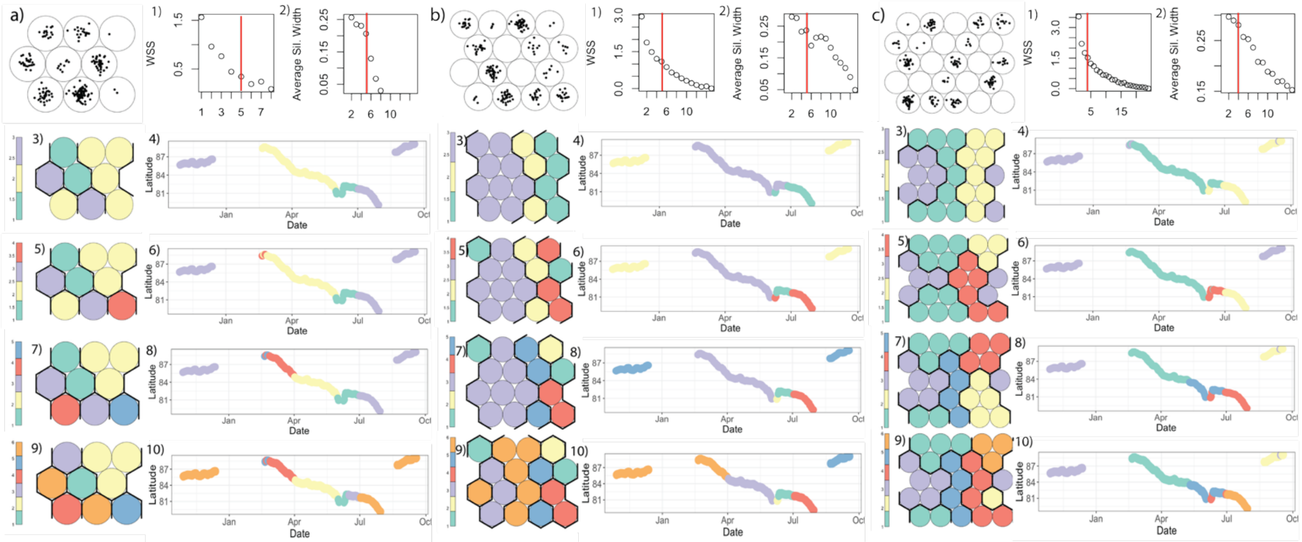

Supplementary Figure 1. Sensitivity analysis regarding Self Organizing Map (SOM) grid size and k-value selection, showing the selection process for a tested grid size of 3x3 (a), 4x4 (b), and 5x5 (c). Within each subsection is plotted the Elbow plot (1; within-clusters sum of squares (WSS) vs. number of clusters (k)), Silhouette plot (2; Average silhouette (Sil) width vs. number of clusters (k)) and the experimental clustering results for a sub-selection of k around the perceived optimums (k = 3, k = 4, k = 5, and k = 6). While all grid cells contained samples at 3x3, clusters were not contiguous across the model grid (e.g. cluster 3 when k = 3, k = 4, and k = 5, cluster 6 when k = 6). At a grid size of 5x5, many of the grid cells did not contain samples indicating too wide of sample spread, and clustering patterns were very similar to those observed on the 4x4 grid for lower k values. Ultimately, a SOM grid of 4x4 as the optimum for sample spread and cluster continuity and k = 5 as the elbow inflection point (WSS) and local maximum for Average Sil. Width (large drop in value at k = 6).

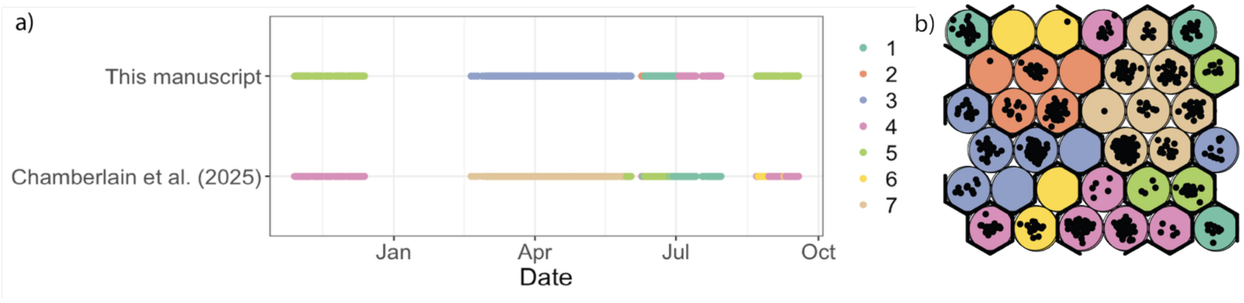

Supplementary Figure 2. All underway DNA samples are presented across the MOSAiC time series (a) colored first by the SOM mode assignments trained using only 11 m underway samples in this manuscript (Fig. S1f), and then by SOM mode assignments trained using a 693 sample full water column dataset including both 11 m underway and 2–4000m CTD water samples (Chamberlain et al. 2025, panel b). The incorporation of additional and shallower surface water samples from the CTD casts results in a more sensitive model and an additional community mode representative of river-influenced water from the Transpolar Drift (Chamberlain et al. 2025). Seasonal modes and their transitions remain otherwise nearly identical between the two model constructions. Due to the greater information provided for training, the Chamberlain et al. (2025) is likely the more accurate representation of community composition, and we recommend using this model in future studies beyond this intentionally simplified methodological comparison.

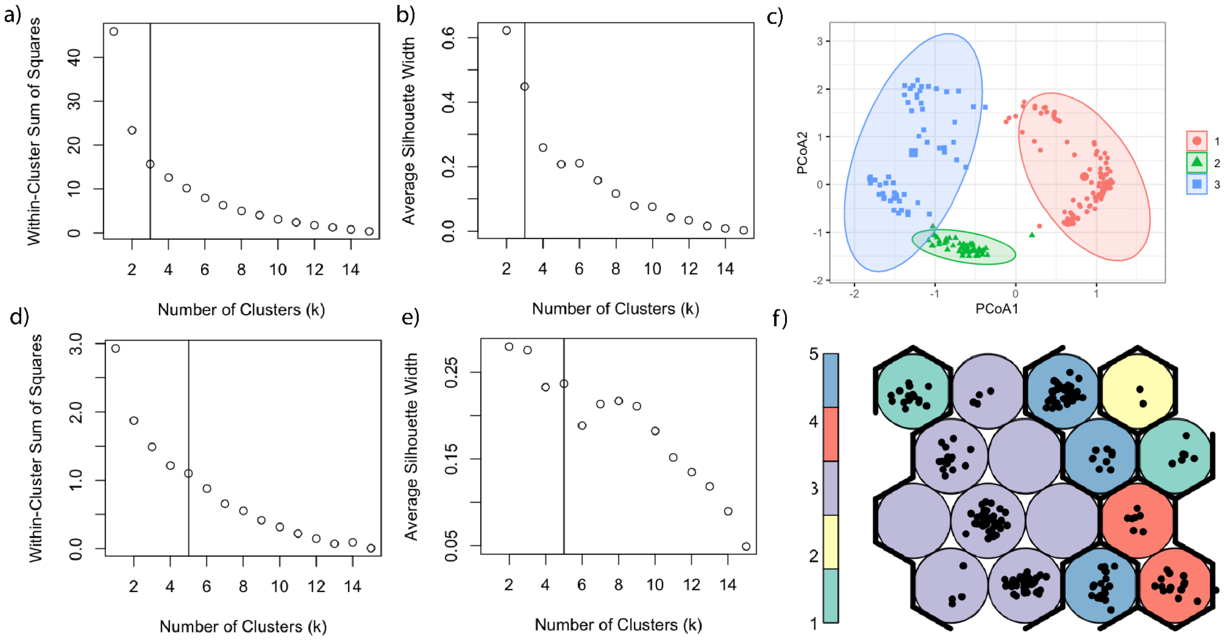

Supplementary Figure 3. The within-cluster sum of squares (a,d) and average silhouette width across increasing *k* values (b,e) for clustering in PCoA space (a,b) and across SOM vectors (d,e). The PCoA ordination (c) shows samples colored and shaped by k-means cluster assignment (k = 3), with

95% confidence ellipses. SOM grid (f) shows map nodes colored by final k-means cluster assignment (k = 5), with black dots representing individual samples.

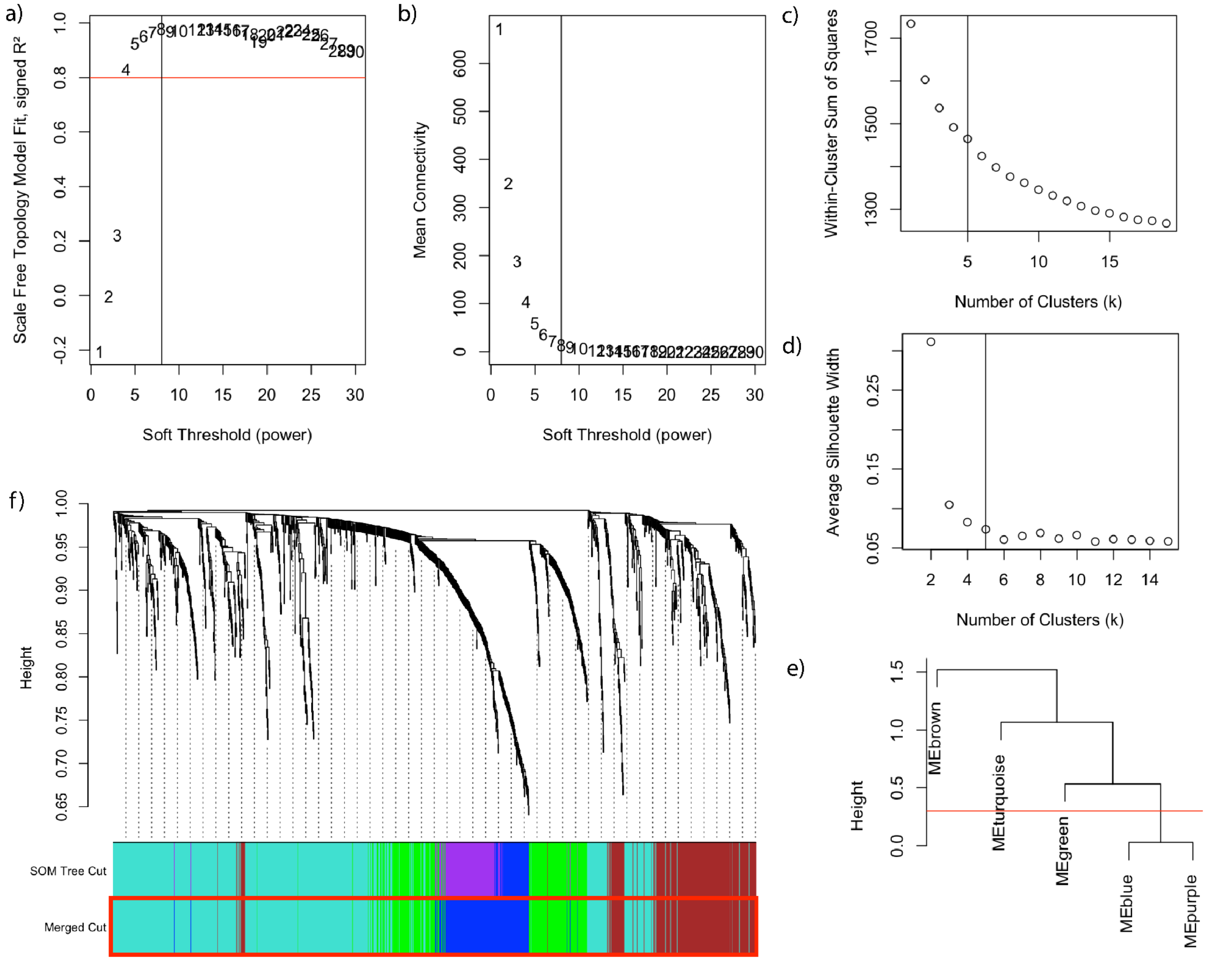

Supplementary Figure 4. Panel (a) shows the scale-free topology model fit (signed R^2) for the WGCNA clustering (ASVs) plotted against soft-thresholding powers, where the red line indicates a threshold of $R^2 = 0.8$. Panel (b) highlights the mean connectivity across the same range of powers. Both a scree plot of within-cluster sum of squares (c) and the average silhouette width (e) were used to determine the optimal number of module clusters (k = 5). To confirm our selection, a clustering dendrogram of the module eigengenes was constructed and branches with high similarity (< 0.3, red line) were merged (final k = 4). The dendrogram of included ASVs (f) indicates module assignment, showing both the original SOM-based module cut and the final selection of merged modules (red box).

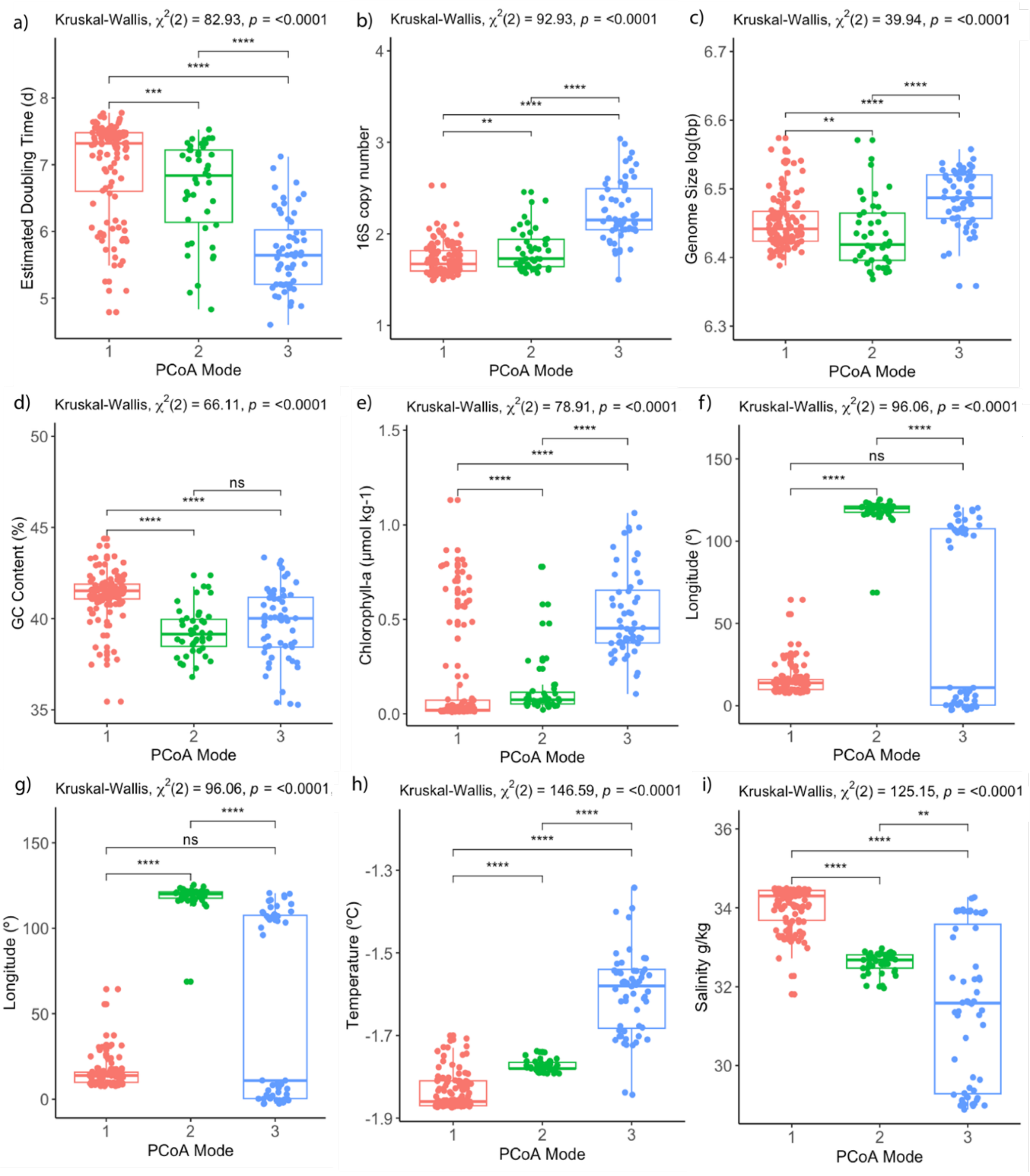

Supplementary Figure 5. Where paired samples were available, SOM cluster is compared to a) gRodon estimated doubling time in hours, b) estimated 16S rRNA gene copy number, c) estimated genome size in bp, logged, d) estimated % GC content, e) chlorophyll-a concentration in μmol kg^{-1}, f) latitude in º, g) longitude in º, h) temperature in ºC, and i) salinity in g kg^{-1}. The horizontal line indicates the median, the box spans the first to the third quartile, the whiskers indicate minimum and maximum values, and the dots indicate all values. The results of a Kruskal-Wallis and post-hoc Wilcoxon-signed rank test comparisons are presented in brackets where ns indicates $p > 0.05$, * indicates $0.01 < p \leq 0.05$, ** indicates $0.001 < p \leq 0.01$, *** indicates $0.0001 < p \leq 0.001$, and **** indicates $p \leq 0.0001$.

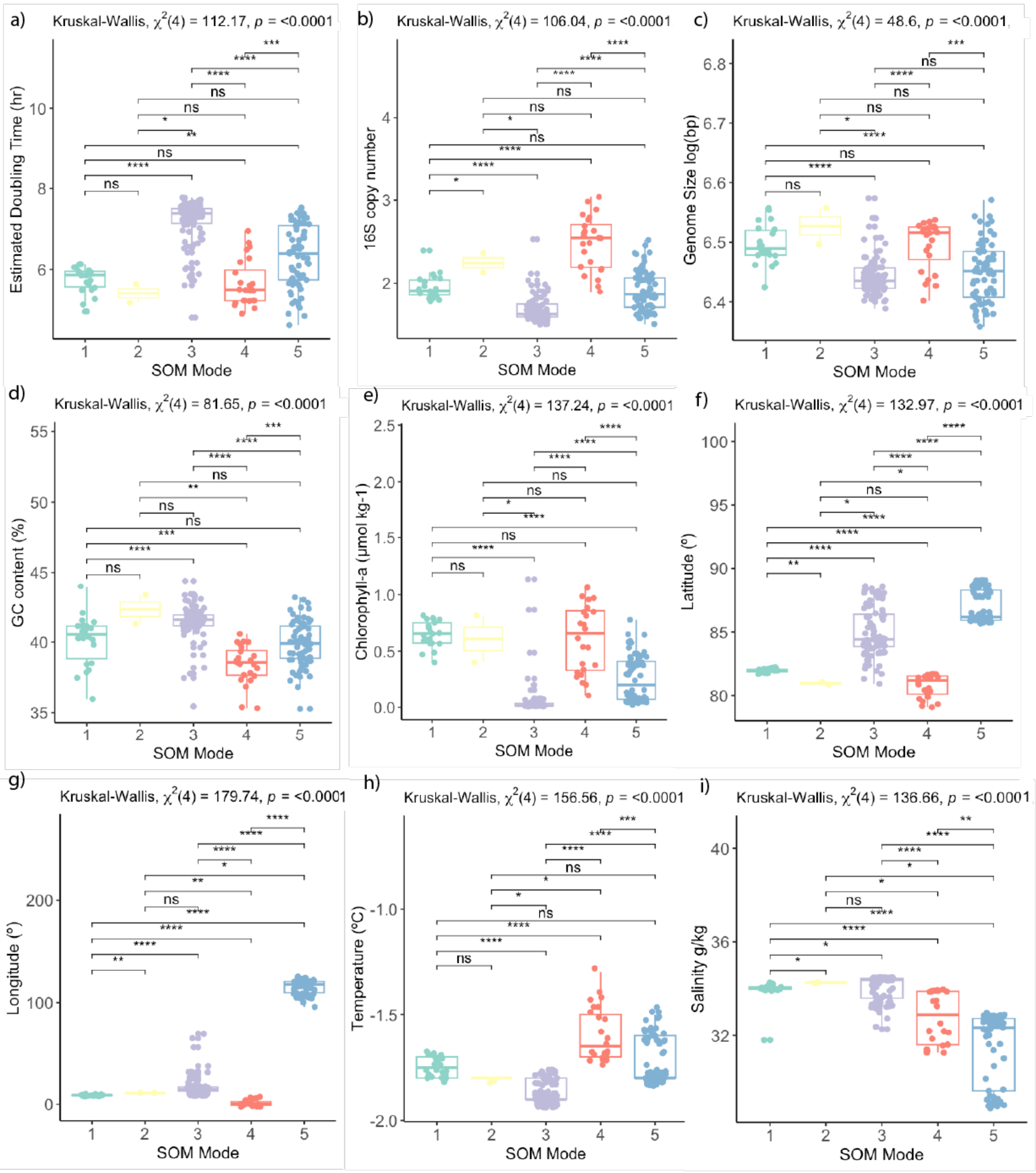

Supplementary Figure 6. Where paired samples were available, SOM cluster is compared to a) gRodon estimated doubling time in hours, b) estimated 16S rRNA gene copy number, c) estimated genome size in bp, logged, d) estimated % GC content, e) chlorophyll-a concentration in µmol kg^{-1}, f) latitude in º, g) longitude in º, h) temperature in ºC, and i) salinity in g kg^{-1}. The horizontal line indicates the median, the box spans the first to the third quartile, the whiskers indicate minimum and maximum values, and the dots indicate all values. The results of a Kruskal-Wallis and post-hoc Wilcoxon-signed rank test comparisons are presented in brackets where ns indicates $p > 0.05$, * indicates $0.01 < p \leq 0.05$, ** indicates $0.001 < p \leq 0.01$, *** indicates $0.0001 < p \leq 0.001$, and **** indicates $p \leq 0.0001$.

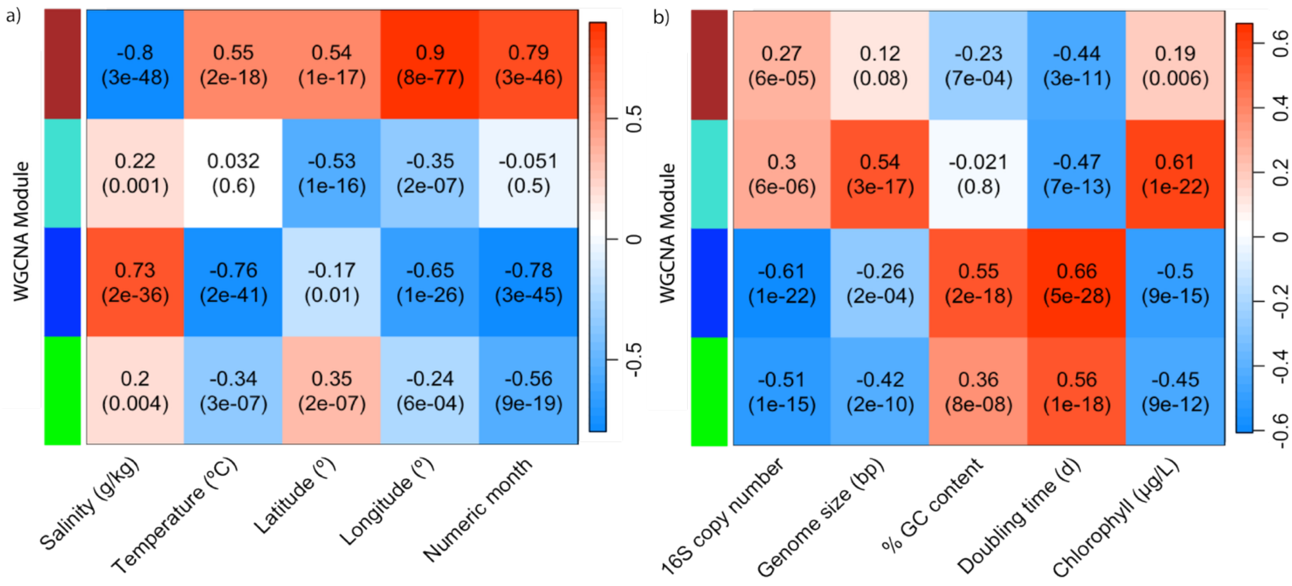

Supplementary Figure 7. The Pearson correlation coefficients between WGCNA module eigengenes and environmental variables (a) or genetic traits (b) are shown as heatmaps. The text in each cell displays the correlation and associated *p*-value and cells are colored by the strength of their correlation (blues indicate negatively correlated relationships while reds indicated positively correlated relationships). Row colors correspond to the WGCNA module assignments of Brown, Blue, Green, and Turquoise.

3 Supplementary References

Chamberlain, Emelia J., Sebastian Rokitta, Björn Rost, Alessandra D'Angelo, Jessie M. Creamean, Brice Loose, Adam Ulfsbo, et al. 2025. "Predictive Links between Microbial Communities and Biological Oxygen Utilization in the Arctic Ocean." *Limnology and Oceanography*. John Wiley & Sons, Ltd: 1–17. doi:10.1002/LNO.70125.
